# Supplementary material for: Deletion of wfs1 Impairs Oligodendrocyte Precursor Cells Dorsal Distribution and Myelination Through the wfs1-hmgcs1 Axis in Zebrafish
Source: Biology (Basel). 2026 Aug 16;15(16):1402. doi: 10.3390/biology15161402 (PMC13510151; doi:10.3390/biology15161402)
Supplement: Supplementary file 1 [file biology-15-01402-s001.zip › File S1-Transcriptome sequencing data and differentially expressed genes (DEGs) in wfs1-- zebrafish at 5 dpf/2.KEGG/wfs1KOvsWT/GSEA/WT/RNA_POLYMERASE/RNA_POLYMERASE(DRE03020).html]

Details for gene set RNA\_POLYMERASE(DRE03020)[GSEA]

|  || Dataset | deg.sample |
| Phenotype | sample.cls |
| Upregulated in class | WT |
| GeneSet | RNA\_POLYMERASE(DRE03020) |
| Enrichment Score (ES) | -0.2975826 |
| Normalized Enrichment Score (NES) | -0.95445514 |
| Nominal p-value | 0.47916666 |
| FDR q-value | 0.67331725 |
| FWER p-Value | 1.0 |
Table: GSEA Results Summary

  

Fig 1: Enrichment plot: RNA\_POLYMERASE(DRE03020)      
 Profile of the Running ES Score & Positions of GeneSet Members on the Rank Ordered List

  

| PROBE | DESCRIPTION (from dataset) | GENE SYMBOL | GENE\_TITLE | RANK IN GENE LIST | RANK METRIC SCORE | RUNNING ES | CORE ENRICHMENT || 1 | 562329 | polr3b |  |  | 1080 | 2.012 | 0.0588 | No |
| 2 | 394162\_1 | polr3f |  |  | 1343 | 1.774 | 0.1324 | No |
| 3 | 325603\_1 | polr1b |  |  | 2801 | 1.083 | 0.1365 | No |
| 4 | 393201 | polr3c |  |  | 3457 | 0.939 | 0.1593 | No |
| 5 | 110438496 | LOC110438496 |  |  | 6681 | 0.521 | 0.0821 | No |
| 6 | 449992 | polr2i |  |  | 8143 | 0.416 | 0.0554 | No |
| 7 | 100003720 | polr2m |  |  | 8893 | 0.375 | 0.0491 | No |
| 8 | 100149268 | polr2b |  |  | 9501 | 0.334 | 0.0455 | No |
| 9 | 541428 | znrd1 |  |  | 9883 | 0.311 | 0.0478 | No |
| 10 | 553733 | polr1e |  |  | 11867 | 0.197 | -0.0053 | No |
| 11 | 100330570 | polr2ea |  |  | 12494 | 0.165 | -0.0174 | No |
| 12 | 553347 | polr2a |  |  | 13422 | 0.119 | -0.0410 | No |
| 13 | 100003720\_1 | polr2m |  |  | 13653 | 0.109 | -0.0432 | No |
| 14 | 796803 | polr2k |  |  | 14176 | 0.084 | -0.0558 | No |
| 15 | 327078 | polr1a |  |  | 14266 | 0.080 | -0.0549 | No |
| 16 | 445412 | polr1d |  |  | 15157 | 0.039 | -0.0810 | No |
| 17 | 572258 | polr3a |  |  | 15372 | 0.029 | -0.0864 | No |
| 18 | 447901 | polr3gla |  |  | 15591 | 0.018 | -0.0924 | No |
| 19 | 554099 | polr2j |  |  | 15654 | 0.014 | -0.0937 | No |
| 20 | 449786 | polr3glb |  |  | 15859 | 0.004 | -0.0999 | No |
| 21 | 445170 | polr2eb |  |  | 16081 | -0.005 | -0.1067 | No |
| 22 | 324088 | polr2gl |  |  | 17039 | -0.034 | -0.1352 | No |
| 23 | 402856 | twistnb |  |  | 17146 | -0.038 | -0.1367 | No |
| 24 | 550263 | polr2h |  |  | 17204 | -0.041 | -0.1366 | No |
| 25 | 792693 | cd3eap |  |  | 17398 | -0.050 | -0.1404 | No |
| 26 | 393257 | polr3d |  |  | 17854 | -0.070 | -0.1515 | No |
| 27 | 334669 | polr2c |  |  | 19911 | -0.162 | -0.2086 | No |
| 28 | 553733\_1 | polr1e |  |  | 21030 | -0.217 | -0.2337 | No |
| 29 | 436588 | polr2d |  |  | 21291 | -0.230 | -0.2312 | No |
| 30 | 569690 | zgc:171971 |  |  | 21603 | -0.246 | -0.2296 | No |
| 31 | 751656 | crcp |  |  | 22983 | -0.326 | -0.2579 | No |
| 32 | 436826 | polr3k |  |  | 23589 | -0.366 | -0.2600 | No |
| 33 | 393538 | polr1c |  |  | 23693 | -0.372 | -0.2461 | No |
| 34 | 336518 | polr3e |  |  | 25079 | -0.453 | -0.2687 | No |
| 35 | 553705 | polr2l |  |  | 25124 | -0.455 | -0.2491 | No |
| 36 | 436817\_1 | polr2f |  |  | 26668 | -0.595 | -0.2702 | Yes |
| 37 | 100000978 | polr3g |  |  | 27176 | -0.644 | -0.2564 | Yes |
| 38 | 553705\_1 | polr2l |  |  | 27280 | -0.647 | -0.2299 | Yes |
| 39 | 436749 | polr3h |  |  | 28141 | -0.762 | -0.2217 | Yes |
| 40 | 562329\_1 | polr3b |  |  | 29745 | -1.146 | -0.2192 | Yes |
| 41 | 325603 | polr1b |  |  | 31528 | -2.998 | -0.1370 | Yes |
| 42 | 394162 | polr3f |  |  | 31586 | -3.218 | 0.0095 | Yes |
Table: GSEA details [plain text format]

  

Fig 2: RNA\_POLYMERASE(DRE03020)      
 Blue-Pink O' Gram in the Space of the Analyzed GeneSet

  

Fig 3: RNA\_POLYMERASE(DRE03020): Random ES distribution      
 Gene set null distribution of ES for **RNA\_POLYMERASE(DRE03020)**
